# Depression and Anxiety in Patients With Cancer: A Cross-Sectional Study

**DOI:** 10.3389/fpsyg.2021.585534

**Published:** 2021-04-15

**Authors:** Abdallah Y. Naser, Anas Nawfal Hameed, Nour Mustafa, Hassan Alwafi, Eman Zmaily Dahmash, Hamad S. Alyami, Haya Khalil

**Affiliations:** ^1^Faculty of Pharmacy, Isra University, Amman, Jordan; ^2^Department of Pharmacy, King Hussein Cancer Center, Amman, Jordan; ^3^Department of Pharmacology and Toxicology, College of Medicine, Umm Al-Qura University, Mecca, Saudi Arabia; ^4^Department of Pharmaceutics, College of Pharmacy, Najran University, Najran, Saudi Arabia

**Keywords:** outpatient, inpatient, Jordan, depression, cancer, anxiety

## Abstract

**Objectives:**

Depression and anxiety persist in cancer patients, creating an additional burden during treatment and making it more challenging in terms of management and control. Studies on the prevalence of depression and anxiety among cancer patients in the Middle East are limited and include many limitations such as their small sample sizes and restriction to a specific type of cancer in specific clinical settings. This study aimed to describe the prevalence and risk factors of depression and anxiety among cancer patients in the inpatient and outpatient settings.

**Materials and Methods:**

A total of 1,011 patients (399 inpatients and 612 outpatients) formed the study sample. Patients’ psychological status was assessed using the Hospital Anxiety and Depression Scale (HADS), the Patient Health Questionnaire (PHQ-9), and the Generalized Anxiety Disorder 7-item (GAD-7) scale. The prevalence rate of depressive and anxious symptomatology was estimated by dividing the number of patients who exceeded the borderline score: 10 or more for each subscale of the HADS scale, 15 or more for the GAD-7 scale, and 15 or more in the PHQ-9 by the total number of the patients. Risk factors were identified using logistic regression.

**Results:**

The prevalence of depressive and anxious symptomatology among all patients was 23.4% and 19.1–19.9%, respectively. Depressive symptomatology was more prevalent across patients who were hospitalized (37.1%) compared with patients in the outpatient setting (14.5%) (*p* < 0.001). Similarly, anxious symptomatology was more prevalent in the inpatient setting (*p* < 0.001). In the inpatient setting, depressive symptomatology was more prevalent among patients with bladder cancer, while severe anxious symptomatology was more prevalent across patients with lung cancer. In the outpatient setting, depressive and anxious symptomatology was more prevalent among breast and prostate cancer patients, respectively. Despite that, around 42.7% and 24.8% of the patients, respectively, reported that they feel anxious and depressed, and only 15.5% of them were using medications to manage their conditions.

**Conclusion:**

Our study findings demonstrated a higher prevalence of depressive and anxious symptomatology in the inpatient setting and advanced disease stages. In addition, the underutilization of antidepressant therapy was observed. There is a need to consider mental disorders as part of the treatment protocol for cancer patients. Enhanced clinical monitoring and treatment of depression and anxiety of cancer patients are required.

## Introduction

In cancer patients, psychological problems such as depression and anxiety persist and can cause an additional burden during their treatment, making it more challenging in terms of its management and control (Walker et al., 2013; Ahmed, 2019), compliance during the treatment course (Ahmed, 2019), duration of hospital stay (Koenig et al., 1992; McDermott et al., 2018), and, ultimately, survival rate (Spiegel et al., 1989; Spiegel and Li, 2007). Previous studies have reported that the prevalence of depressive disorders among cancer patients is two to three times higher than those of the general population (Massie, 2004; Pirl, 2004). Previous studies that evaluated psychological distress among cancer patients have reported various heterogeneous prevalence rates that differed according to clinical settings (outpatient clinics, hospital settings, and palliative care), stage of the disease (newly diagnosed, recurrence, survivorship, or advanced stages), and phase of treatment (Mitchell et al., 2011; Krebber et al., 2014; Caruso et al., 2017), which ranged between 5.0 and 49.0% (Walker et al., 2013). A previous meta-analysis that explored the prevalence of depression among cancer patients and included 211 studies (representing more than 82,000) reported a different prevalence rate of depression—one that differed by the type of instrument, type of cancer, and treatment phase (Krebber et al., 2014). In addition, the prevalence rate of depressive disorders among cancer patients differed by cancer site and ranged from 5.6% for patients with genitourinary cancer to 13.1% for patients with lung cancer (Walker et al., 2014). Furthermore, depression is more common among patients with severe illness and advanced stages of malignancy (Kaasa et al., 1993; Delgado-Guay et al., 2009). A previous critical review that included 11 previous systematic reviews and meta-analyses, which aimed to identify risk factors of depression among cancer patients, has reported a wide range of factors that play a key role in developing depression besides the biological factors (the type of cancer, stage of the disease, and treatment-related factors) (Caruso et al., 2017). This includes individual factors (family history, personal psychiatric history, and personality traits) and interpersonal and social factors (a history of stressful life events, loneliness, social isolation, low-socioeconomic status, and lack of social support) (Caruso et al., 2017). Assessment of psychological distress among cancer patients is important in order to recognize patients who need help and further assessment and, therefore, subsequent healthcare intervention. This increasingly highlights the fact that depression is a substantial problem in cancer patients.

In 2018, a total of 10,898 new cancer cases were diagnosed in Jordan within a population of 9,903,798. An age-standardized incident rate was 157.8 per 100,000, while the age-standardized mortality rate was 89.7 per 100,000. The top five most prevalent cancers were breast cancer, lung cancer, colorectal cancer, bladder cancer, and leukemia (World Health Organization, 2018). Even though depression is a significant complication of cancer and its occurrence is higher than in the general population (Centre for Disease Control and Prevention, 2019), it is often neglected. Studies on the prevalence of depression and anxiety among cancer patients in the Middle East are limited, with many limitations such as small sample size and being restricted to a specific type of cancer in specific clinical settings (Al Ahwal et al., 2014; Abou Kassm et al., 2018; Ahmadi Gharaei et al., 2019). The primary aim of this study is to describe the prevalence and risk factors of depressive and anxious symptomatology in cancer patients in the inpatient and outpatient settings. Additionally, we aimed to explore the pattern of use of antidepressants among the study participants. This will enable us to identify cancer patients who are at higher risk of depression and anxiety. Mental and medical support can be directed to cancer patients who are at higher risk in order to control their psychological problems and improve their clinical outcome. Early identification is critical in the management of depressive symptoms and plays an important role in improving their adherence to the therapy and the overall control of the disease.

## Materials and Methods

### Study Design

This was a cross-sectional study conducted at the King Hussein Cancer Center (KHCC) in Amman, Jordan, between October 2019 and February 2020. According to the last available statistics in 2015, KHCC provides medical care to around 60.0% of all cancer patients in Jordan (Abdel-Razeq et al., 2015). KHCC is the main hospital and the single specialized tertiary hospital in Jordan and provides comprehensive clinical management and cancer care to adults and pediatric patients from Jordan and the surrounding region.

### Sampling Strategy

Data were collected from the inpatient and outpatient settings using a convenience sampling technique. Cancer patients who have any type of cancer from any stage and who are willing to participate in the study formed the study population. The inclusion criteria were (a) patients aged 18 years and above with a confirmed cancer diagnosis and (b) patients who had no apparent cognitive deficit. Patients were excluded if they were (a) below 18 years of age and (b) unable to participate in this study due to physical or emotional distress. This is due to the difficulties in detecting depression in cancer patients with emotional distress because of patients’ reluctance to discuss their emotional well-being (Krebber et al., 2014). Eligible patients were identified and assessed by a clinical pharmacist (NM). Recruitment of patients was conducted by two pharmacists (AH and NM). For patients who agreed to participate, the study’s aim and objectives were explained thoroughly. Information sheets were provided to the patients for further clarification about the study. In addition, patients were informed that their agreement to participate in the study is considered as written consent. Patients’ clinical data were obtained from their medical charts in collaboration with a clinical pharmacist at the cancer center.

### Sample Size

The target sample size was estimated based on the WHO recommendations for the minimal sample size needed for a prevalence study (Lwanga and Lemeshow, 1991). Using a confidence interval of 95%, a standard deviation of the prevalence rate of 0.5, a margin of error of 5%, we determined that the required sample size was 385 patients.

### Depression and Anxiety Assessment Scales

Previously validated assessment scales, the Patient Health Questionnaire (PHQ)-9, Generalized Anxiety Disorder 7-item (GAD-7) scale, and Hospital Anxiety and Depression Scale (HADS) were used to assess depressive and anxious symptomatology among the study participants. These screening instruments were frequently used and validated as brief screening tools among cancer patients for depressive and anxious symptomatology (Härter et al., 2006; Sawaya et al., 2016; Terkawi et al., 2017). These assessment scales provide a symptomatological assessment based on predefined cut-off points.

Different assessment scales were used to fit the inpatient and outpatient settings, as recommended by previous literature. The HADS and GAD-7 instruments were previously validated to be used for hospitalized patients (secondary care settings) in multiple previous studies, including studies on patients with cancer (Zigmond and Snaith, 1983; Bjelland et al., 2002; Hartung et al., 2017; Esser et al., 2018). In contrast, the PHQ-9 and GAD-7 instruments were recommended for patients in primary care settings (Hinz et al., 2016; Esser et al., 2018; Levis et al., 2019). The PHQ-9 scale is a nine-question instrument given to patients in a primary-care setting to screen for the presence and severity of depressive symptomatology (Hartung et al., 2017). This instrument was used to assess depressive symptomatology among cancer patients in the outpatient setting, and the GAD-7 instrument was used to screen for anxiety among them (Esser et al., 2018). In the inpatient setting, the HADS instrument was used, which is a 14-question instrument given to patients in a secondary-care setting to screen for the presence and severity of depressive and anxious symptomatology (Hartung et al., 2017).

The use of a pre-existing scale has the advantage of using a validated and tested instrument, which increases the reliability of its measure. The PHQ-9 and the GAD-7 instruments ask the patients about the degree of applicability of each item (question), using a 4-point Likert scale. Patients’ responses ranged from 0 to 3, where 0 means “Not at all” and 3 means “Nearly every day.” The HADS instrument is a 14-question questionnaire that asks the patients about the degree of applicability of each item (question), using a 4-point Likert scale. Patients’ response ranges from 0 to 3, where 0 means “Often” and 3 means “Very seldom” or from “Not at all” to “Most of the time.”

### Methods of Analysis

#### An Estimate of Prevalence and Classification of Depression and Anxiety

Prevalence rates of depressive and anxious symptomatology were determined using a cut-off point as recommended by the authors of the PHQ-9, GAD-7, and HADS scale. At the inpatient setting using the HADS instrument, depressive and anxious symptomatology were defined as a total score of (10 or more) at “depression subscale” or “anxiety subscale,” respectively (Zigmond and Snaith, 1983). At the outpatient setting, depressive symptomatology was defined as a total score of 15 and above in the PHQ-9 instrument, indicating a case with moderately severe or severe depression (Kroenke et al., 2001). Anxious symptomatology was defined using the GAD-7 instrument with a total score of 15 and above, indicating a case with severe anxious symptomatology (Spitzer et al., 2006). The higher the score, the more severe the case identified by any scale.

The prevalence rate of depressive symptomatology was estimated by dividing the number of patients who exceeded the borderline score by the total number of patients. The same procedure was followed to calculate the prevalence rate of anxious symptomatology in the inpatient and the outpatient settings.

#### At The Inpatient Setting

The HADS instrument was used in the inpatient setting, which includes two subscales (anxiety and depression) with seven items for each. Items are scored from 0 to 3, generating a total score ranging from 0 to 21 on each subscale. A total score of 0–7 indicates a normal case, 8–10 a borderline case, and 11–21 an abnormal case of depressive or anxious symptomatology according to the subscale score (Zigmond and Snaith, 1983). In addition, anxious symptomatology was assessed using the GAD-7 scale as a second measure and results were compared with HADS.

#### At The Outpatient Setting

The PHQ-9 instrument was used in the outpatient setting, which includes nine items. Items are scored from 0 to 3, generating a total score ranging from 0 to 27. A total score of 0–4 indicates minimal depression, 5–9 mild depression, 10–14 moderate depression, 15–19 moderately severe depression, and 20–27 severe depression (Schwenk et al., 2011). The GAD-7 instrument includes seven items. Items are scored from 0 to 3, generating a total score ranging from 0 to 21. A total score of 5–9 indicates mild anxiety, 10–14 moderate anxiety, and 15–21 severe anxiety (Spitzer et al., 2006).

### Statistical Analysis

Descriptive statistics were used to describe patients’ demographic characteristics, medication use, and comorbidities. Continuous data were reported as mean ± SD. Categorical data were reported as percentages (frequencies). Logistic regression was used to estimate odds ratios (ORs), with 95% confidence intervals (CIs) for anxious or depressive symptomatology. Logistic regression models were carried out using anxious or depressive symptomatology scores above the cut-off points as highlighted in the section “Materials and Methods.” The cut-off point for the PHQ-9 and GAD-7 scale that was used to identify severe depressive symptomatology and severe anxious symptomatology in the outpatient setting was 15 and above. The cut-off for the HADS scales that was used to identify depressive symptomatology and anxious symptomatology in the inpatient setting was 10 and above (whether for the depression or anxiety subscale). A two-sided *p* < 0.05 was considered statistically significant. The statistical analyses were carried out using SPSS for Windows (version 25).

## Results

### Patient Characteristics

Out of 1,041 patients who were approached during the study period, a total of 1,011 patients (response rate of 97.1%) participated in the study: inpatient setting = 399, outpatient setting = 612. Table 1 details the baseline characteristics of the patients in the inpatient and outpatient settings. The mean age of the patients was 54.9 years (±15.2). The majority of patients (*n* = 560, 55.4%) were males, married (*n* = 833, 82.4%), and unemployed (*n* = 471, 46.6%), with an income of 500 JD or below (*n* = 737, 72.9%). Around 18.4% (*n* = 186) of the patients reported that their cancer was metastatic. More than half (*n* = 566, 56.0%) of the patients reported being treated with chemotherapy.

**TABLE 1:** The baseline characteristics of the patients in the inpatient and the outpatient settings.

**Demographics**	**Overall (*n* = 1,011)**	**Inpatient (*n* = 399)**	**Outpatient (*n* = 612)**	***P*-value**
**Gender No. (%)**				
Male	560 (55.4%)	225 (56.4%)	335 (54.7%)	0.861
**Age** (years, mean, and SD)	54.9 (15.2)	55.6 (± 15.6)	54.4 (± 15.0)	0.245
**Marital status No. (%)**				
Single	99 (9.8%)	37 (9.3%)	62 (10.1%)	0.935
Married	833 (82.4%)	331 (83.2%)	502 (82.0%)	0.672
Divorced	23 (2.3%)	8 (2.0%)	15 (2.5%)	0.830
Widowed	55 (5.4%)	22 (5.5%)	33 (5.4%)	1.000
**Employment status No. (%)**				
Employed	276 (27.3%)	103 (25.8%)	173 (28.3%)	**0.014**
Unemployed	471 (46.6%)	172 (43.1%)	299 (48.9%)	0.082
Retired	264 (26.1%)	124 (31.1%)	140 (22.9%)	**0.004**
**Income No. (%)**				
Lower than 500 JD**	737 (72.9%)	327 (82.0%)	410 (67.0%)	**0.000**
500 to 1000 JD**	197 (19.5%)	54 (13.5%)	143 (23.4%)	**0.000**
1000 to 1500 JD**	35 (3.5%)	7 (1.8%)	28 (4.6%)	**0.021**
1500 JD** or above	42 (4.2%)	11 (2.8%)	31 (5.1%)	0.078
**Cancer metastasis No. (%)**				
Yes	186 (18.4%)	80 (20.1%)	106 (17.3%)	0.116
No	445 (44.0%)	146 (36.6%)	299 (48.9%)	
Don’t know	380 (37.6%)	173 (43.4%)	207 (33.8%)	
**Stage of metastasis (*n* = 80 in the inpatient, *n* = 106 in the outpatient) No. (%)**				
Stage 1	10 (5.4%)	2 (2.5%)	8 (7.5%)	0.331
Stage 2	18 (9.7%)	5 (6.3%)	13 (12.3%)	0.343
Stage 3	25 (13.4%)	8 (10.0%)	17 (16.0%)	0.537
Stage 4	133 (71.5%)	65 (81.3%)	68 (64.2%)	**0.005**
Cancer therapy* No. (%)				
Surgery	40 (4.0%)	40 (10.0%)	0	**0.000**
Chemotherapy	566 (56.0%)	190 (47.6%)	376 (61.4%)	**0.000**
Combination of surgery and chemotherapy	352 (34.8%)	119 (29.8%)	233 (38.1%)	**0.007**
Radiotherapy	298 (29.5%)	116 (29.1%)	182 (29.8%)	0.832
Don’t receive treatment (on palliative therapy)	36 (3.6%)	36 (9.0%)	0	**0.000**
**Do you think or feel that you feel anxious? No. (%)**				
Yes	432 (42.7)	220 (55.1)	212 (34.6)	**0.000**
**Do you think or feel that you feel depressed? No. (%)**				
Yes	251 (24.8)	148 (37.1)	103 (16.8)	**0.000**
**Are you currently using or have you ever used antidepressant? (*n* = 148 in the inpatient; *n* = 103 in the outpatient) No. (%)**				
Yes	39 (15.5)	21 (14.1)	18 (17.5)	0.126
**Category of use for the antidepressant medications: (*n* = 20 in the inpatient; *n* = 11 in the outpatient)**				
Sertraline	9 (29.0)	2 (10.0)	7 (63.6)	
Citalopram	5 (16.1)	5 (25.0)	0	
Mirtazapine	2 (6.5)	2 (10.0)	0	
Fluoxetine	2 (3.2)	1 (5.0)	1 (9.1)	
Amitriptyline	1 (3.2)	1 (5.0)	0	
Escitalopram	1 (3.2)	0	1 (9.1)	
Fluvoxamine	1 (3.2)	0	1 (9.1)	
Paroxetine	1 (3.2)	0	1 (9.1)	
**Age first consumed antidepressant (years): (*n* = 20 in the inpatient; *n* = 11 in the outpatient)**	52.2 (± 17.4)	50.4 (± 18.7)	54.6 (± 15.9)	0.550
**Used in the last year? (*n* = 20 in the inpatient; *n* = 11 in the outpatient)**				
Yes	16 (51.6)	10 (50.0)	6 (54.5)	0.689
**Currently using antidepressants? (*n* = 20 in the inpatient; *n* = 11 in the outpatient)**				
Yes	21 (67.7)	12 (60.0)	9 (81.8)	1.000
**Medical monitored? (*n* = 20 in the inpatient; *n* = 11 in the outpatient)**				
Yes	25 (80.6)	14 (70.0)	11 (100.0)	1.000
**Consumption reason (more than one choice could be chosen): (*n* = 20 in the inpatient; *n* = 11 in the outpatient)**				
Anxiety	11 (35.5)	5 (25.0)	6 (54.5)	
Depression	26 (83.9)	15 (75.0)	11 (100.0)	
**Have you received instruction on how to use the antidepressant therapy? (*n* = 21 in the inpatient; *n* = 18 in the outpatient)**				
Yes	36 (92.3)	19 (90.5)	17 (94.4)	0.366
**If yes, who gave you these instructions? (*n* = 19 in the inpatient; *n* = 17 in the outpatient)**				
Physicians	34 (94.4)	18 (94.7)	16 (94.1)	
Nurse	1 (2.8)	1 (5.3)	0	
Pharmacist	1 (2.8)	0	1 (5.9)	
**Do you consider these instructions important? (*n* = 19 in the inpatient; *n* = 17 in the outpatient)**				
Yes	27 (75.0)	15 (78.9)	12 (70.6)	0.546

*Patient could select more than one type of therapy; SD, standard deviation.

**JD, Jordanian Dinar (equal 1.41$). Bold values indicates that the p-value is statistically significant and that there was statistically significant difference between the inpatient and outpatient settings.

The most common cancer type in the study was blood cancer (*n* = 196, 19.3%), followed by colorectal cancer (*n* = 178, 17.6%) and lung cancer (*n* = 120, 11.9%). Please refer to Supplementary Table 1, which shows the distribution of cancer types among study participants.

### Use of Antidepressants

Around 42.7% (*n* = 432) and 24.8% (*n* = 251) of the patients reported that they feel anxious and depressed, respectively, and only 15.5% (*n* = 39) of them were using antidepressants. The two most commonly used antidepressant medications were sertraline (29.0%, *n* = 9) and citalopram (16.1%, *n* = 5).

The mean age of the patients when they started using antidepressants was 52.2 years (±17.4). Around 51.6% (*n* = 16) of these patients were using antidepressants last year, and 67.7% (*n* = 21) are using it currently. The majority of the patients (80.6%, *n* = 25) were using antidepressant medications under medical supervision. The main indication for the use of antidepressants was to alleviate depression for 83.9% (*n* = 26), and 35.5% (*n* = 11) were using them to alleviate anxiety. Around 92.3% (*n* = 36) of the patients reported that they had received instructions on how to use antidepressants. The main source of these instructions was a physician (94.4%, *n* = 34). The majority of the patients (75.0%, *n* = 27) believe that these instructions are important (Table 1).

The main reasons patients consider the healthcare professionals’ instructions important include the notion that they increase the safety (27.3%, *n* = 6) and effectiveness (27.3%, *n* = 6) of the medication; they decrease side effects and drug interactions (27.3%, *n* = 6) and increase confidence in therapy (18.2%, *n* = 4). Nine patients reported that they have questions about antidepressants, and they were mainly considering treatment side effects, mechanisms of action, and treatment time (Supplementary Table 2).

Supplementary Table 3 highlights characteristics of antidepressant use and patient knowledge. More than half of the patients (56.8%, *n* = 21) reported that they have increased the dose of antidepressant medication without consulting the doctor. Around 40.5% (*n* = 15) of the patients reported that they were experiencing side effects from the use of antidepressants. The main three side effects were nausea, dizziness, and insomnia, suffered by 73.3%, 60.0%, and 40.0% of patients, respectively. About 29.7% (*n* = 11) and 21.6% (*n* = 8) of the patients reported that they think that antidepressant use can cause addiction and tolerance, respectively. More than half of the patients (59.5%, *n* = 22) reported that antidepressant therapy should gradually be withdrawn at the end of the treatment. When the patients were asked about whether they had stopped using antidepressant without consulting the doctor, 32.4% (*n* = 12) reported they had. The two most common reasons for this practice were improved depressive symptoms (50.0%, *n* = 6) and low tolerance of side effects of the medication (41.7%, *n* = 5).

### Prevalence of Depression and Anxiety

The prevalence of depressive symptomatology among all patients was 23.4% (*n* = 237; 89 from the outpatient setting and 148 from the inpatient setting). Depressive symptomatology was more prevalent in the inpatient setting (37.1%; *n* = 148) compared to the outpatient setting (14.5%; *n* = 89). The prevalence of anxious symptomatology among all patients was 19.1% (*n* = 193) using the HADS for the inpatients or 19.9% (*n* = 201), using the GAD-7 for the inpatients. Similarly, anxious symptomatology was more prevalent in the inpatient setting, at 35.6% (*n* = 142), using the HADS, or 37.6% (*n* = 150) (using the GAD-7). Table 2 below details the prevalence of depressive and anxious symptomatology among the patients stratified by severity.

**TABLE 2:** Prevalence of depression and anxiety among the patients’ stratified by severity.

	**Settings**
**Outpatient settings (*n* = 612)**	
	**The severity of depression (using PHQ-9)**
Minimal depression	282 (46.1%)
Mild depression	165 (27.0%)
Moderate depression	76 (12.4%)
Moderately severe depression	56 (9.2%)
Severe depression	33 (5.4%)
	**The severity of anxiety (using GAD-7)**
Normal	339 (55.4%)
Mild anxiety	142 (23.2%)
Moderate anxiety	80 (13.1%)
Severe anxiety	51 (8.3%)
**Inpatient settings (*n* = 399)**	
	**The severity of depression (using HADS – depression subscale)**
Normal	195 (48.9%)
Abnormal borderline case	56 (14.0%)
Abnormal case	148 (37.1%)
	**The severity of anxiety**
*Using HADS- anxiety subscale*	
Normal	208 (52.1%)
Abnormal borderline case	49 (12.3%)
Abnormal case	142 (35.6%)
*Using GAD-7*	
Mild anxiety	168 (42.1%)
Moderate anxiety	81 (20.3%)
Severe anxiety	150 (37.6%)

*HADS, Hospital Anxiety and Depression Scale; GAD, Generalized Anxiety Disorder.*

Table 3 below details the prevalence of depressive and anxious symptomatology stratified by severity and type of cancer in the inpatient and the outpatient setting. In the inpatient setting, depressive symptomatology was more common across patients with bladder cancer, and severe anxious symptomatology was more prevalent among patients with lung cancer. In the outpatient setting, depressive symptomatology was more common among patients with breast cancer, and the highest prevalence of anxious symptomatology was among patients with prostate cancer.

**TABLE 3:** Prevalence of depression and anxiety stratified by type of cancer and severity.

	**Type of cancer**														
	**The severity of depression (using PHQ-9)**														
		**Colorectal cancer**	**Head and neck cancer**	**Blood cancer**	**Lung cancer**	**Breast cancer**	**Cervical cancer**	**Bladder cancer**	**Ovarian cancer**	**Stomach cancer**	**Bone marrow cancer**	**Pancreas cancer**	**Prostate cancer**	**Liver cancer**	**Brain cancer**
Outpatient settings (n = 612)	Minimal depression	74 (57.8)	10 (50.0)	59 (45.7)	30 (39.5)	15 (42.9)	12 (34.3)	11 (47.8)	9 (39.1)	9 (37.5)	8 (47.1)	7 (36.8)	7 (46.7)	9 (60.0)	4 (33.3)
	Mild depression	32 (25.0)	4 (20.0)	42 (32.6)	19 (25.0)	5 (14.3)	6 (17.1)	6 (26.1)	7 (30.4)	10 (41.7)	3 (17.6)	7 (36.8)	3 (20.0)	4 (26.7)	4 (33.3)
	Moderate depression	11 (8.6)	4 (20.0)	10 (7.8)	10 (13.2)	5 (14.3)	10 (28.6)	5 (21.7)	5 (21.7)	2 (8.3)	3 (17.6)	3 (15.8)	2 (13.3)	0	3 (25.0)
	Moderately severe depression	7 (5.5)	1 (5.0)	10 (7.8)	13 (17.1)	5 (14.3)	6 (17.1)	1 (4.3)	3 (13.0)	0	2 (11.8)	2 (10.5)	0	1 (6.7)	1 (8.3)
	Severe depression	4 (3.1)	1 (5.0)	8 (6.2)	4 (5.3)	5 (14.3)	1 (2.9)	0	0	3 (12.5)	0	0	3 (20.0)	1 (6.7)	1 (8.3)
	**The severity of anxiety (using GAD-7)**														
	Mild anxiety	22 (17.2)	7 (35.0)	34 (26.4)	20 (26.3)	10 (28.6)	9 (25.7)	4 (17.4)	7 (30.4)	3 (12.5)	6 (35.3)	5 (26.3)	2 (13.3)	3 (20.0)	3 (25.0)
	Moderate anxiety	10 (7.8)	3 (15.0)	14 (10.9)	14 (18.4)	5 (14.3)	8 (22.9)	2 (8.7)	4 (17.4)	3 (12.5)	2 (11.8)	2 (10.5)	2 (13.3)	3 (20.0)	3 (25.0)
	Severe anxiety	6 (4.7)	1 (5.0)	13 (10.1)	5 (6.6)	6 (17.1)	5 (14.3)	2 (8.7)	1 (4.3)	3 (12.5)	1 (5.9)	0	3 (20.0)	1 (6.7)	1 (8.3)
Inpatient settings (*n* = 399)	**The severity of depression (using HADS- depression subscale)**														
	Normal	25 (50.0)	11 (52.4)	36 (53.7)	16 (36.4)	23 (48.9)	6 (60.0)	8 (44.4)	7 (41.2)	7 (50.0)	5 (27.8)	8 (53.3)	8 (61.5)	6 (46.2)	7 (50.0)
	Abnormal borderline case	6 (12.0)	0	9 (13.4)	7 (15.9)	8 (17.0)	3 (30.0)	1 (5.6)	3 (17.6)	4 (28.6)	1 (5.6)	1 (6.7)	1 (7.7)	3 (23.1)	3 (21.4)
	Abnormal case	19 (38.0)	10 (47.6)	22 (32.8)	21 (47.7)	16 (34.0)	1 (10.0)	9 (50.0)	7 (41.2)	3 (21.4)	5 (27.8)	6 (40.0)	4 (30.8)	5 (38.5)	4 (28.6)
	**The severity of anxiety**														
	*Using HADS – anxiety subscale*														
	Normal	28 (56.0)	11 (52.4)	43 (64.2)	17 (38.6)	25 (53.2)	4 (40.0)	9 (50.0)	8 (47.1)	7 (50.0)	4 (22.2)	8 (53.3)	10 (76.9)	8 (61.5)	6 (42.9)
	Abnormal borderline case	5 (10.0)	1 (4.8)	8 (11.9)	4 (9.1)	7 (14.9)	4 (40.0)	2 (11.1)	2 (11.8)	4 (28.6)	2 (11.1)	1 (6.7)	0	2 (15.4)	4 (28.6)
	Abnormal case	17 (34.0)	9 (42.9)	16 (23.9)	23 (52.3)	15 (31.9)	2 (20.0)	7 (38.9)	7 (41.2)	3 (21.4)	5 (27.8)	6 (40.0)	3 (23.1)	4 (30.8)	4 (28.6)
	*Using GAD-7*														
	Mild anxiety	25 (50.0)	11 (52.4)	35 (52.2)	10 (22.7)	22 (46.8)	4 (40.0)	7 (38.9)	7 (41.2)	8 (57.1)	2 (11.1)	5 (33.3)	7 (53.8)	5 (38.5)	5 (35.7)
	Moderate anxiety	8 (16.0)	1 (4.8)	13 (19.4)	9 (20.5)	7 (14.9)	1 (10.0)	5 (27.8)	5 (29.4)	3 (21.4)	5 (27.8)	6 (40.0)	3 (23.1)	3 (23.1)	2 (14.3)
	Severe anxiety	17 (34.0)	9 (42.9)	19 (28.4)	25 (56.8)	18 (38.3)	5 (50.0)	6 (33.3)	5 (29.4)	3 (21.4)	4 (22.2)	4 (26.7)	3 (23.1)	6 (46.2)	7 (50.0)

*HADS, Hospital Anxiety and Depression Scale; GAD, Generalized Anxiety Disorder.*

### Risk Factors of Depression and Anxiety

In the inpatient setting, logistic regression analysis identified the following groups as being at a higher risk of depressive symptomatology: a) patients with metastatic cancer, OR: 2.62 (95% CI 1.61–4.28) and b) patients at an advanced stage of the disease, stage 3, OR: 5.26 (95% CI 1.05–26.41) and stage 4, OR: 2.73 (95% CI 1.61–4.62). In the outpatient setting, patients with metastatic cancer were the only group that showed a statistically significant increased risk of depressive symptomatology, OR: 3.36 (95% CI 1.33–8.50), compared with others.

Regarding anxious symptomatology, in the inpatient setting the following groups were identified to be at a higher risk using the HADS: a) patients with metastatic cancer, OR: 2.10 (1.29–3.42) and b) patients at stage four of the disease, OR: 2.39 (95% CI 1.42–4.04). On the other hand, patients who are treated with a combination of chemotherapy and surgery showed a lower risk of anxious symptomatology, OR: 0.54 (95% CI 0.33–0.86). On the basis of the GAD-7 scale, the only patient group that showed a higher risk of anxious symptomatology was the group with metastatic cancer, OR: 2.23 (95% CI 1.32–3.74). In the outpatient setting, unemployed patients—OR: 1.89 (95% CI 1.03–3.35)—and patients with metastatic cancer—OR: 2.47 (95% CI 1.15–5.33)—were at higher risk of anxious symptomatology (Table 4).

**TABLE 4:** Logistic regression analysis.

**Variable**	**Inpatient settings**			**Outpatient settings**	
	**Odds ratio (95% CI) for depression according to HADS**	**Odds ratio (95% CI) for anxiety according to HADS**	**Odds ratio (95% CI) for severe anxiety according to GAD-7**	**Odds ratio (95% CI) for severe depression according to PHQ-9**	**Odds ratio (95% CI) for severe anxiety according to GAD-7**
**Gender**					
Male (Reference)	1.00	1.00	1.00	1.00	1.00
Female	1.12 (0.75–1.68)	1.21 (0.80–1.82)	1.43 (0.91–2.26)	1.29 (0.64–2.59)	1.64 (0.92–2.90)
**Age**					
Less than 50 years	1.00	1.00	1.00	1.00	1.00
50 years and above	0.76 (0.50–1.18)	1.03 (0.66–1.60)	0.87 (0.54–1.42)	0.81 (0.39–1.65)	0.68 (0.38–1.21)
**Marital status**					
Single (Reference)	1.00	1.00	1.00	1.00	1.00
Married	1.15 (0.66–1.99)	1.15 (0.66–2.00)	0.83 (0.45–1.50)	0.80 (0.34–1.90)	1.03 (0.48–2.17)
Divorced	1.73 (0.43–7.01)	1.85 (0.46–7.50)	3.29 (0.81–13.40)	–	0.78 (0.10–6.07)
Widowed	0.63 (0.24–1.63)	0.52 (0.19–1.44)	1.21 (0.46–3.18)	1.14 (0.26–4.99)	0.33 (0.04–2.47)
**Occupational status**					
Employed (Reference)	1.00	1.00	1.00	1.00	1.00
Unemployed	1.15 (0.76–1.73)	1.35 (0.90–2.04)	1.37 (0.86–2.17)	1.45 (0.71–2.95)	1.89 (1.03–3.35)*
Retired	1.16 (0.75–1.80)	1.05 (0.67–1.63)	0.89 (0.54–1.47)	0.74 (0.30–1.83)	0.81 (0.39–1.66)
**Income**					
Below 500 JD (Reference)	1.00	1.00	1.00	1.00	1.00
500 to 1000 JD	0.68 (0.36–1.27)	0.81 (0.44–1.50)	0.68 (0.33–1.42)	1.05 (0.46–2.39)	0.68 (0.32–1.44)
1000 to 1500 JD	1.28 (0.28–5.79)	1.37 (0.30–6.19)	0.52 (0.06–4.38)	1.37 (0.31–6.05)	1.90 (0.63–5.72)
More than 1500 JD	0.97 (0.28–3.37)	0.67 (0.18–2.57)	0.70 (0.15–3.27)	0.57 (0.08–4.33)	0.35 (0.05–2.65)
**Duration of disease**					
Less than 12 years (Reference)	1.00	1.00	1.00	1.00	1.00
12 years and above	1.10 (0.72–1.67)	0.71 (0.47–1.08)	0.85 (0.53–1.37)	1.42 (0.68–2.95)	1.35 (0.74–2.46)
**Metastasis**					
No (Reference)	1.00	1.00	1.00	1.00	1.00
Yes	2.62 (1.61–4.28)***	2.10 (1.29–3.42)**	2.23 (1.32–3.74)**	3.36 (1.33–8.50)*	2.47 (1.15–5.33)*
**Stage of cancer**					
1 (Reference)	1:00	1.00	1.00	1:00	1.00
2	0.42 (0.05–3.79)	1.21 (0.20–7.33)	0.79 (0.09–7.13)	3.33 (0.71–15.69)	3.44 (0.92–12.94)
3	5.26 (1.05–26.41)*	0.60 (0.12–3.00)	1.92 (0.45–8.20)	1.10 (0.14–8.55)	2.44 (0.68–8.80)
4	2.73 (1.61–4.62)***	2.39 (1.42–4.04)**	–	2.29 (0.95–5.49)	1.30 (0.56–3.02)
**Cancer therapy**					
Don’t receive treatment (Reference)	1.00	1.00	1.00	1.00	1.00
Chemotherapy	1.12 (0.74–1.67)	1.11 (0.74–1.67)	1.13 (0.72–1.80)	0.79 (0.39–1.62)	0.85 (0.47–1.53)
Combination of surgery and chemotherapy	0.77 (0.49–1.20)	0.54 (0.33–0.86)*	0.84 (0.50–1.40)	1.28 (0.62–2.62)	1.19 (0.66–2.14)
Radiotherapy	1.51 (0.97–2.34)	1.10 (0.70–1.72)	1.48 (0.91–2.41)	1.90 (0.92–3.90)	1.01 (0.54–1.90)
Surgery	1.02 (0.52–2.00)	1.55 (0.80–3.00)	1.06 (0.50–2.25)	–	–

*p < 0.05, **p < 0.01, ***p < 0.001.

*HADS, Hospital Anxiety and Depression Scale; GAD, Generalized Anxiety Disorder; PHQ, Patient Health Questionnaire.*

## Discussion

This study aimed to identify the prevalence of depressive and anxious symptomatology among cancer patients and to identify key risk factors using validated assessment tools. Additionally, we explored the pattern of use of antidepressants among the study participants. Several dimensions were investigated in this study, including patients’ characteristics, the prevalence of anxious and depressive symptomatology according to the type of cancer and treatment settings (inpatient versus outpatient). Our findings showed that the prevalence of depressive and anxious symptomatology among cancer patients was 23.4% and 19.1–19.9%, respectively. Increased likelihood of depressive and anxious symptomatology was detected among patients in the inpatient setting (37.1% and 35.6–37.6%, respectively). Screening of frequently prescribed anxiolytics and antidepressants was investigated, revealing that for the most part, SSRIs were prescribed, but as low as 15.5% of depressed and anxious patients received the required treatment (Waraich et al., 2004; Brothers et al., 2011; Findley et al., 2012; Li et al., 2012; Baltenberger et al., 2014; Nakash et al., 2014; Jassim et al., 2015; van den Berg et al., 2015; Kanera et al., 2016; Lengacher et al., 2016; Reich et al., 2017; Ahmed, 2019).

Our research employed two validated tools (GAD-7 and HADS) to assess the prevalence of anxiety among cancer patients in the inpatient setting, and both of them were reliable and showed a significant correlation (correlation coefficient: 0.812) in terms of the prevalence of anxiety (37.6% versus 35.6% in the inpatient setting). The increased cancer-specific depressive symptomatology was noted across settings (inpatient and outpatient) and was significantly higher in the inpatient settings.

Several factors may impact the development of depression and anxiety among cancer patients, including the cancer type, stage, grade, and treatment option (Smith, 2015). Interestingly, our results are aligned with the findings of several studies, where specific tumor types can lead to depression and anxiety, particularly head and neck, lung, breast, and prostate cancer (Pitman et al., 2018), which, in our research, showed that depression and anxiety are more prevalent in the inpatient setting in patients with head and neck cancer, lung cancer, and bladder cancer, while in the outpatient setting, they were more prevalent among patients diagnosed with prostate and breast cancer.

In addition to the type of cancer, the treatment option impacted the prevalence of anxiety and depression among cancer patients. Cancer treatments that entail chemotherapy may induce depression through specific biological mechanisms. Furthermore, the literature reported that antiemetic medications, steroids, and androgen suppression therapy (for prostate cancer) were reported to induce depression (Smith, 2015; Ismail et al., 2017; Nead et al., 2017; Niedzwiedz et al., 2019). The findings of our study revealed that most cancer patients receive chemotherapy alone (56.0%) or chemotherapy and surgery (34.8%), thus increasing their risk of depression and/or anxiety. The use of chemotherapy-induced nausea and vomiting (CINV) medications, as well as steroids, are a mainstay for the prevention and treatment of CINV (Rao and Faso, 2012) and, consequently, contribute to the high prevalence of anxiety and depression among cancer patients.

### Antidepressant Use Patterns

This study also explored the pattern of use of antidepressants among cancer patients stratified by type, where the rate of using antidepressants among patients diagnosed with depression was as low as 15.5%. Low use of antidepressant therapy is an alarming sign, especially for cancer patients who are receiving specialized cancer services (Waraich et al., 2004; Findley et al., 2012; Nakash et al., 2014). Antidepressants should be introduced as soon as the patient is diagnosed with depression. Use of antidepressants should be individualized according to the patient’s health profile to address his or her symptoms. The choice of antidepressants should be based on the patient’s concurrent medications, history, and symptoms (Ahmed, 2019). Pharmacological therapy is recommended for patients with severe depression, while patients with mild to moderately severe depression are recommended to receive psychotherapy. Several behavioral approaches can be implemented to improve the psychological status of cancer patients, such as cognitive behavioral therapy, mindfulness-based approaches, and self-management strategies. Management of depression and treatment initiation should be under medical supervision, due to the nature of antidepressants and the high probability of drug-drug interaction, adverse effects, and the need for dose adjustment (Ahmed, 2019).

The majority of patients were prescribed selective serotonin reuptake inhibitor (SSRI) antidepressants (sertraline, citalopram, fluoxetine, fluvoxamine, and paroxetine), while tetracyclic antidepressants (mirtazapine) and tricyclic antidepressants (amitriptyline) were prescribed to a lesser extent. The selection of an anxiolytic or antidepressant medication needs to be made under clear guidelines that consider interactions with chemotherapeutic and other concurrently administered medication, as well as side effects, to enable identification of specific contraindications. Sertraline and citalopram are usually recommended for depression and anxiety treatment, as they have the least tendency for interactions and are usually well tolerated (Chochinov, 2001; Smith, 2015). Although the use of medications for depression and anxiety among cancer patients in this study was low, the selection of pharmacological agents is in line with the above recommendations. However, a patient-centered approach and a customized treatment plan need to be considered, as studies reported that the SSRIs need to be avoided in elderly patients due to the risk of hyponatremia. Fluoxetine and paroxetine are contraindicated in patients being treated with tamoxifen. Furthermore, mirtazapine should be avoided where white blood cells are compromised and SSRIs are to be avoided where platelets are compromised (Pitman et al., 2018). Finally, since almost 70.0% of the patients reported that they experienced nausea, SSRIs need to be avoided as this will augment chemotherapy-induced nausea and vomiting.

### Risk Factors of Depression and Anxiety

Our findings suggest that severe depressive and anxious symptomatology are substantially more common in patients with cancer in the inpatient than in the outpatient setting. This significant difference could be attributed to the severity of the disease, where hospitalized treatment of cancer patients is mainly employed for the management of acute phases, initial onsets, severe cases, or late stages. A previous study involving more than 5,000 patients has reported that more symptoms of anxiety are associated with cancer within the inpatient setting and in patients in the advanced stages, whereas patients at early stages demonstrated lower anxiety symptoms. Furthermore, disease stage was associated with depression, particularly in men (Vodermaier et al., 2011).

Further analysis using logistic regression was conducted to identify patients at risk of developing depressive and anxious symptomatology. Similar to reported data, the prevalence of anxiety and depression among women is higher than in men (Smith, 2015). As discussed earlier, the advancement of the stage of the disease has an impact on depression and anxiety. Patient vulnerability exacerbates the ability to become depressed and anxious, which is reported to be caused by some socioeconomic factors and disease stages (Chochinov, 2001; Smith, 2015). Such results align with our findings about patients with metastasis, advanced disease stages, and lower-income were more vulnerable to developing anxiety and/or depression. Such findings support the need to consider mental disorders as part of the treatment protocol for cancer patients and calls for enhanced clinical monitoring and treatment of depression and anxiety symptoms among cancer patients.

### Strengths and Limitations

To the best of our knowledge, this is the first and largest study in the Middle East region to investigate the prevalence of depressive and anxious symptomatology and use of antidepressants among cancer patients without restriction on the type of cancer or clinical settings. Previous studies have focused on a specific type of cancer (breast cancer and colorectal cancer) (Al Ahwal et al., 2014; Abou Kassm et al., 2018; Ahmadi Gharaei et al., 2019) and had a small sample size (not more than 100 patients) (Al Ahwal et al., 2014; Abou Kassm et al., 2018).

We have explored the prevalence of depressive and anxious symptomatology among cancer patients without any restriction on the age, gender, duration of the disease, or treatment phase. Our broad inclusion criteria have enabled us to explore the difference in severity of depressive and anxious symptomatology among different demographic groups and across different stages of the treatment and the course of illness. This study has many strengths that increase its value and reliability: (a) using validated assessment tools for depressive and anxious symptomatology, (b) anxious symptomatology was assessed using two assessment tools (HADS and GAD-7), and both of them showed consistent findings, (c) employing a large sample size, (d) not restricting the inclusion criteria for the specific type of cancer or specific settings (inpatients or outpatients), which increased the generalizability of our findings, and (e) our exclusion criteria minimized the risk of deriving imprecise information (related to the patients’ psychological status) from any suspected physical or emotional distress. On the other hand, this study has limitations: (a) the study design itself, a cross-sectional study design, limited our ability to identify causality between study variables, as it is only capable of showing an association between variables, and (b) the sample size of a few cancer subgroups was small due to a small population in this category nationwide. This might affect our ability to determine the prevalence of depressive and anxious symptomatology among patients with specific types of cancer, especially types for which we have a very low number of patients, such as head and neck cancer; (c) the use of convenience sampling techniques might affect the generalizability of our findings as a prevalent study and may introduce sampling bias (which might not precisely represent the targeted population); (d) the use of different assessment tools to describe the prevalence of depressive symptomatology between the inpatient and the outpatient settings might not provide a fair comparison; (e) there is a lack of non-responder data; and (f) the antidepressant medication information is based on small subsamples making conclusions difficult to sustain. Therefore, our findings should be interpreted carefully.

## Conclusion

Our study demonstrated a higher prevalence of both depressive and anxious symptomatology within the inpatient setting and advanced stages of the disease. There is a need for cancer management clinical guidelines to consider early assessment and management of depression and anxiety and to continue to monitor it throughout treatment.

## Data Availability Statement

The original contributions presented in the study are included in the article/Supplementary Material, further inquiries can be directed to the corresponding author/s.

## Ethics Statement

Approval for this study was obtained from the Institutional Review Board Committee at King Hussein Cancer Centre in Jordan (No. 19 KHCC 94). For patients who agreed to participate, the study’s aim and objectives were explained thoroughly. Information sheets were provided to the patients for further clarification about the study. In addition, patients were informed that their agreement to participate in the study is considered as a written consent.

## Author Contributions

AN contributed to the study design and did the data analysis. AN, AH, NM, and HK conducted the study and collected the data. AN, ED, NM, and HA wrote the first draft of the article. AN, HA, ED, HA, and NM were involved in interpretation of the data. All authors reviewed the manuscript for important intellectual content, provided final approval of the version to be published, and agreed to be accountable for all aspects of the work in ensuring that questions related to the accuracy or integrity of any part of the work are appropriately investigated and resolved.

## Conflict of Interest

The authors declare that the research was conducted in the absence of any commercial or financial relationships that could be construed as a potential conflict of interest.

## Supplementary Material

Supplementary Table 1 Type of cancer among the patient stratified by settings.

Supplementary Table 2 Questions about antidepressants treatment.

Supplementary Table 3 Characteristics of antidepressants utilization and patients’ knowledge about them.
