# Supplementary material for: Depression and Anxiety in Patients With Cancer: A Cross-Sectional Study
Source: Front Psychol. 2021 Apr 15;12:585534. doi: 10.3389/fpsyg.2021.585534 (PMC8081978; doi:10.3389/fpsyg.2021.585534)
Supplement: Supplementary Table 1 — Type of cancer among the patient stratified by settings. [file Table_1.docx]

**Table S1 Type of cancer among the patient stratified by settings.**

| **Type of cancer** | **Overall** | **Inpatient settings** | **Outpatient settings** |
| --- | --- | --- | --- |
|  | Frequency (%) | Frequency (%) | Frequency (%) |
| **Colorectal cancer** | **178 (17.6)** | **50 (12.5)** | **128 (20.9)** |
| Colon | 144 (14.2) | 44 (11.0) | 100 (16.3) |
| Colorectal | 18 (1.8) | 3 (0.8) | 15 (2.5) |
| Rectal | 16 (1.6) | 3 (0.8) | 13 (2.1) |
| **Blood cancer** | **196 (19.3)** | **67 (16.8)** | **129 (21.1)** |
| Lymphoma | 148 (14.6) | 60 (15.0) | 88 (14.4) |
| Leukaemia | 24 (2.4) | 1 (0.3) | 23 (3.8) |
| Multiple myeloma | 24 (2.4) | 6 (1.5) | 18 (2.9) |
| **Head and neck cancer** | **41 (4.1)** | **21 (5.3)** | **20 (3.3)** |
| Pharyngeal | 13 (1.3) | 9 (2.3) | 4 (0.7) |
| Larynx | 12 (1.2) | 6 (1.5) | 6 (1.0) |
| Nasopharynx | 8 (1.0) | 0 | 8 (1.3) |
| Mouth | 6 (1.0) | 4 (1.0) | 2 (0.3) |
| Jaw | 2 (0.2) | 2 (0.5) | 0 |
| **Other types of cancer** | **595 (58.9)** | **261 (65.4)** | **334 (54.5)** |
| Lung | 120 (11.9) | 44 (11.0) | 76 (12.4) |
| Breast | 82 (8.1) | 47 (11.8) | 35 (5.7) |
| Cervical | 46 (4.5) | 10 (2.5) | 35 (5.7) |
| Bladder | 41 (4.1) | 18 (4.5) | 23 (3.8) |
| Ovarian | 40 (4.0) | 17 (9.8) | 23 (3.8) |
| Stomach | 38 (3.8) | 14 (3.5) | 24 (3.9) |
| Bone marrow | 35 (3.5) | 18 (4.5) | 17 (2.8) |
| Pancreas | 34 (3.4) | 15 (3.8) | 19 (3.1) |
| Prostate | 28 (2.8) | 13 (3.3) | 15 (2.5) |
| Liver | 28 (2.8) | 13 (3.3) | 15 (2.5) |
| Brain | 26 (2.6) | 14 (3.5) | 12 (2.0) |
| Kidney | 19 (1.9) | 10 (2.5) | 9 (1.5) |
| Sarcoma | 17 (1.7) | 8 (2) | 9 (1.5) |
| Oesophagus | 12 (1.2) | 2 (0.5) | 10 (1.6) |
| Thyroid | 12 (1.2) | 9 (2.3) | 3 (0.5) |
| Testicular | 10 (1.0) | 6 (1.5) | 4 (1.2) |
| Bone | 4 (0.4) | 1 (0.3) | 3 (0.5) |
| Thymus | 2 (0.2) | 1 (0.3) | 1 (0.2) |
| Adrenal gland | 1 (0.1) | 1 (0.3) | 0 |
