# Supplementary material for: Depression and Anxiety in Patients With Cancer: A Cross-Sectional Study
Source: Front Psychol. 2021 Apr 15;12:585534. doi: 10.3389/fpsyg.2021.585534 (PMC8081978; doi:10.3389/fpsyg.2021.585534)
Supplement: Supplementary Table 2 — Questions about antidepressants treatment. [file Table_2.docx]

**Table S2: Questions about antidepressants treatment.**

| **Question raised by the patients** | **Overall (n = 9)** | **Inpatient (n = 2)** | **Outpatient (n = 7)** |
| --- | --- | --- | --- |
| Side effects. | 3 (33.3) | 2 (100.0) | 1 (14.3) |
| Mechanism of action of the drug. | 2 (22.2) | 0 | 2 (28.6) |
| Treatment Time. | 2 (22.2) | 1 (50.0) | 1 (14.3) |
| Interaction with other drugs. | 1 (11.1) | 1 (50.0) | 0 |
| Start time of drug action. | 1 (11.1) | 0 | 1 (14.3) |
| If the medication causes dependence | 1 (11.1) | 0 | 1 (14.3) |
