# Supplementary material for: Depression and Anxiety in Patients With Cancer: A Cross-Sectional Study
Source: Front Psychol. 2021 Apr 15;12:585534. doi: 10.3389/fpsyg.2021.585534 (PMC8081978; doi:10.3389/fpsyg.2021.585534)
Supplement: Supplementary Table 3 — Characteristics of antidepressants utilization and patients’ knowledge about them. [file Table_3.docx]

**Table S3: Characteristics of antidepressants utilization and patients’ knowledge about them.**

| Variable | Overall | Inpatient | Outpatient |
| --- | --- | --- | --- |
| **Have you ever increased your dose without consulting your doctor?** (n= 20 in the inpatient; n= 17 in the outpatient) | | | |
| Yes | 21 (56.8) | 18 (90.0) | 3 (17.6) |
| **Are you experiencing side effects from the use of antidepressants?** (n= 20 in the inpatient; n= 17 in the outpatient) | | | |
| Yes | 15 (40.5) | 9 (45.0) | 6 (35.3) |
| **If so, what effect (s)** (more than one choice could be chosen) (n= 9 in the inpatient; n= 6 in the outpatient) | | | |
| Nausea | 11 (73.3) | 6 (30.0) | 5 (83.3) |
| Dizziness | 9 (60.0) | 8 (40.0) | 1 (16.7) |
| Insomnia | 6 (40.0) | 0 | 6 (100.0) |
| Headache | 5 (33.3) | 0 | 5 (63.3) |
| Vomiting | 4 (26.7) | 4 (20.0) | 0 |
| Anxiety | 2 (13.3) | 2 (10.0) | 0 |
| Diarrhea | 2 (13.3) | 2 (10.0) | 0 |
| Weight gain | 2 (13.3) | 0 | 2 (33.3) |
| Decrease libido | 1 (6.7) | 0 | 1 (16.7) |
| **Can antidepressants cause addiction?** (n= 20 in the inpatient; n= 17 in the outpatient) | | | |
| Yes | 11 (29.7) | 7 (35.0) | 4 (23.5) |
| **Can antidepressants cause tolerance?** (n= 20 in the inpatient; n= 17 in the outpatient) | | | |
| Yes | 8 (21.6) | 5 (25.0) | 3 (17.6) |
| **Should the drug be withdrawn at the end of the treatment gradually?** (n= 20 in the inpatient; n= 17 in the outpatient) | | | |
| Yes | 22 (59.5) | 10 (50.0) | 12 (70.6) |
| **Did you stop treatment without consulting the doctor?** (n= 20 in the inpatient; n= 17 in the outpatient) | | | |
| Yes | 12 (32.4) | 5 (25.0) | 7 (41.2) |
| **If so, for what reason (s)** (n= 5 in the inpatient; n= 7 in the outpatient)**?** | | | |
| Improvement of depressive symptoms. | 6 (50.0) | 1 (20.0) | 5 (71.4) |
| Low tolerance to side effects. | 5 (41.7) | 5 (100.0) | 0 |
| Interactions with other medicines. | 2 (16.7) | 1 (20.0) | 1 (14.3) |
| **Do you use other medication (s) besides the antidepressant?** (n= 20 in the inpatient; n=17 in the outpatient) | | | |
| Yes | 13 (35.1) | 7 (35.0) | 6 (35.3) |
| **If so, what are these medications?** (more than one choice could be chosen) (n= 7 in the inpatient; n= 6 in the outpatient) | | | |
| Propranolol | 6 (46.2) | 3 (42.9) | 3 (50.0) |
| Lorazepam | 5 (38.5) | 3 (42.9) | 2 (33.3) |
| Carbamazepine | 3 (23.1) | 1 (14.3) | 2 (33.3) |
| Warfarin | 2 (15.4) | 1 (14.3) | 1 (16.7) |
| Alprazolam | 1 (7.7) | 0 | 1 (16.7) |
